# Testing Extended Parallel Processing Model in the Korean COVID-19 Context: Effect of Moral Intuitions as Moderators

**DOI:** 10.3389/fpubh.2021.756281

**Published:** 2021-11-02

**Authors:** Changhyun Ahn, Ghee Young Noh

**Affiliations:** ^1^Health and New Media Research Institute, Hallym University, Chuncheon-Si, South Korea; ^2^Media School, Hallym University, Chuncheon-Si, South Korea

**Keywords:** COVID, morality, fear, self-efficacy, moral foundation theory, EPPM

## Abstract

Despite the possible social implications of the coronavirus disease 2019 (COVID-19), previous studies of the extended parallel processing model (EPPM) in the context of COVID-19 overlooked the emotional aspects when processing fear-inducing COVID-19-related messages. Drawing upon the moral foundation theory (MFT), this study aimed to (a) apply EPPM in the Korean COVID-19 context, (b) introduce MFT and explain why moral intuitions can be related to the processing of COVID-19 messages, and (c) examine the moderating role of moral intuitions in the EPPM model. Based on the theoretical backgrounds, this study tested EPPM hypotheses and also tested whether moral intuition can moderate the relationship between perceived self-efficacy, perceived threat, fear of COVID-19, and health compliance behavioral intention. This study conducted an online survey using measurements of perceived self-efficacy, perceived threat, MFQ-20, fear of COVID, and health compliance. Our study showed three main findings. First, our study found the main effects of (a) self-efficacy on health compliance behavioral intention and (b) perceived threat on health compliance behavioral intention. Second, our study found that morality moderated the main effects of self-efficacy or perceived threat and also moderated EPPM interaction on fear of COVID. Third, the moderation of morality in the relationship between self-efficacy and health compliance behavioral intention showed that health compliance intention decreased as morality increased. Our findings suggest that people can consider COVID-19 as a social and moral issue that involves protecting others.

## Introduction

As the coronavirus disease 2019 (COVID-19) global pandemic worsened, previous studies applied and examined the extended parallel processing model (EPPM) by Witte (1) in the COVID-19 context (2–4). Although previous studies on EPPM have found some important moderating variables, these studies overlook the role of morality in the context of EPPM and COVID-19. As Kim and Chung (5) noted, people might perceive COVID-19-related messages and behavioral intentions as a moral issue that involves their concerns about infecting and harming others. It was also suggested that people in collectivist societies, such as East Asia and South Korea, could especially perceive COVID-19 as a moral issue, rather than just a disease (5). However, previous studies on EPPM did not consider morality at all and also they did not consider it as a moderator when applying EPPM in the COVID-19 context.

One of the challenges in considering morality in the EPPM and COVID-19 context entails how to define morality and measure it for use in quantitative research. In this regard, moral foundation theory (MFT) (6) offers a useful theoretical and methodological tool that integrates morality in the COVID-19 context. Specifically, MFT suggests that human moral issues generally involve five discrete moral intuitions: care, fairness, loyalty, authority, and purity. These moral intuitions have been conceptualized and validated as quantifiable measurement tools that can be used and applied to various human moral issues (7).

Drawing upon the theory of EPPM and MFT, this study aims to (a) apply EPPM in the Korean COVID-19 context, (b) introduce MFT and explain why moral intuitions can be related to the processing of COVID-19 messages, and (c) examine the moderating role of moral intuitions in the EPPM model.

## Extended Parallel Processing Model

The EPPM argues that evaluating a fear-inducing message initiates either fear control or danger control processing (1, 8). It also explained that the type of processing that occurs depends on the perceived self-efficacy and perceived threat. This means that fear control processing occurs when individuals perceive the fear induced by a message as greater than self-efficacy and are consequently more likely to control the fear itself, rather than critically accept the message; therefore, they would more likely reject the message (fear control processing). Conversely, danger control processing occurs when individuals perceive self-efficacy as greater than the fear induced by the message, therefore, they would more likely accept the message (danger control processing). In short, EPPM proposes that fear control processing occurs in low efficacy/high threat conditions (LE/HT), and danger control processing occurs in high efficacy/low threat conditions (HE/LT).

Previous studies have applied EPPM in the COVID-19 context across different countries, such as East Asia (3, 4, 9), North America (10–12), and the Middle East (2, 13, 14). These studies proposed various unique variables that relate to applying EPPM to the COVID-19 context, such as age, gender, education, economic status, work experience, risk information exposure, intention to follow government recommendations, willingness to work, and self-esteem.

Although recent studies suggest that COVID-19 situations may involve morality (5, 15–17), it has not been tested as a moderator in EPPM. In the next section, we introduce MFT and argue how morality can be involved in the psychological processing of COVID-19 situations.

## Moral Foundation Theory

In essence, morality entails judging which specific human actions are good or bad; such judgment is based on moral standards with which people can generally agree [(18), p. 119–120]. The MFT suggests that humans developed discrete categories of morality to distinguish between good and bad, which can be generally applied across cultures through human evolution (19). Specifically, such discrete morality generally involves five moral domains: care, fairness, loyalty, authority, and purity (20). Based on the idea of discrete moral domains, MFT argues that humans judge actions as good or bad using moral domains (care, fairness, loyalty, authority, purity), which are both innate and learned and vary across cultures [(6), p. 68].

Although the application of MFT heavily depends on each context, conceptualizing the five moral domains from the MFT offers some useful insights into why COVID-19 situations might involve morality issues. In COVID-19 contexts, care violation involves infecting loved ones due to not wearing masks or maintaining social distancing; fairness violation involves people who were in close contact with COVID-19-confirmed patients, but ignore the mandatory self-quarantine period; loyalty violation involves not staying at home while other community members are in lockdown situations; authority violation involves not following the COVID-19 policies of the governments and acting upon individual beliefs or false infodemic news; purity violation involves the intentional gathering of a crowd of people, even when such actions are unnecessary for survival.

Few studies have empirically tested how the five moral intuitions can be related to emotional responses or COVID-related behavioral intentions (5, 15, 21, 22). However, these studies showed mixed results. For example, Chan (15) found that care and fairness were significant predictors of COVID-19-behavioral intentions, while loyalty had a marginal effect on wearing masks and social distancing. In the context of East Asian countries, Triandis (17) explained that moral transgression in collectivist cultures, such as Korea and Japan, might depend on the communal, autonomy, and divinity codes; violating these codes may arouse contempt, anger, and disgust because of loyalty, authority, and purity domain violation [(16, 17), p. 916]. Regarding COVID-19 and moral violation in a collectivist society, Kim and Chung (5) revealed how the loyalty and authority domain in collectivist cultures is used to judge moral actions, as it shows an example of a society where mask wearing has become an autonomous communal code and not wearing a mask is considered as betraying communal expectation. This study also found that many South Koreans considered going to crowded entertainment places as an immoral act, especially during the COVID-19 pandemic. Qian and Yahara (22) conducted a cross-sectional survey using 1,856 Japanese samples; however, this study found that only care and authority were significant predictors of COVID-19 preventive behaviors. In contrast to Eastern countries, Harper et al. (21) found that all moral intuitions, except authority (i.e., care, fairness, loyalty, and purity), significantly correlated with behavioral change and fear of COVID-19.

In short, previous studies have shown how moral intuition can be related to the fear of COVID-19 and behavioral intentions. These studies suggested that the psychological processing of COVID-19-related messages can be more than the dynamics between perceived self-efficacy and threat; rather, the processing of COVID-19 messages can involve another layer, which is moral intuition. However, it is still unknown how and whether moral intuition can serve as a moderator if morality is combined with the EPPM. Therefore, this study aims to (a) apply EPPM in the Korean COVID-19 context, (b) examine whether moral intuition can moderate the effect of perceived efficacy, threat, fear, and behavioral intentions.

Hypothesis (H)1. Self-efficacy will negatively predict fear of COVID-19.
H2. Self-efficacy will positively predict health compliance behavioral intention.
H3. The threat will positively predict fear of COVID-19.
H4. The threat will negatively predict health compliance behavioral intention.
H5. Self-efficacy and threat interact with fear of COVID-19 such that participants in the HE/LT (danger control: high efficacy/low threat) conditions will show less fear than participants in the LE/HT (fear control: low efficacy/high threat) conditions.
H6. Self-efficacy and threat interact with health compliance behavioral intention so that participants in the HE/LT (danger control: high efficacy/low threat) conditions will show higher health compliance behavioral intention than participants in the LE/HT (fear control: low efficacy/high threat) conditions.
Research question 1(RQ1). Can moral intuition moderate the relationship between self-efficacy, threat, fear of COVID-19, and health compliance behavioral intention?
RQ2. What is the relationship between moral intuition, fear of COVID-19, and health compliance behavioral intention?

## Method

### Participants and Procedure

Samples were collected from December 16, 2020, to December 22, 2020, in South Korea, using panel sampling (*n* = 1,500). The panel was based on age, gender, geographic region, and political orientation. A professional research company was used to conduct an online survey; 132,842 emails were sent and 2,371 people volunteered to participate. At the beginning of the online survey, participants were informed that their responses would be anonymous and informed that they are agreeing to participate by clicking and filling in responses. Volunteer participants were randomly assigned to read a mock-up personal COVID-19 story, and then all participants completed survey measurements. The final data comprised 1,500 people, with a response rate of 63.3%. All measurements and survey materials were written in Korean and approved by an institutional review board in a University in South Korea.

### Stimulus

Based on previous studies on EPPM, this study created two mock-up stories (high vs. low involvement) of a perfectly healthy person in their early 30's, who had been wearing masks but unfortunately caught up with COVID-19. The mock-up stimulus comprised about 334 words; high vs. low conditions were manipulated based on psychological and geographic distance (high—patient from South Korea, low—patient from Montenegro).

#### Permission to Reuse and Copyright

Figures, tables, and images will be published under a Creative Commons CC-BY license and permission must be obtained for use of copyrighted material from other sources (including re-published/adapted/modified/partial figures and images from the internet). It is the responsibility of the authors to acquire the licenses, to follow any citation instructions requested by third-party rights holders, and to cover any supplementary charges.

### Measurements

#### EPPM Measurements

Extended parallel processing model measurements were adopted from Witte's (23). Risk-Behavior-Diagnosis (RBD) scale and modified to fit the COVID-19 situation to measure self-efficacy and threat. Self-efficacy was measured using six items (e.g., “wearing a mask is effective for COVID-19 prevention”) based on a 5-point scale (1—strongly disagree, 5—strongly agree) of the modified RBD scale (Cronbach's α = 0.90, *M* = 4.42, Mdn = 4.50, SD = 0.56); threat was measured using six items (e.g., “COVID-19 is a serious threat”) based on a 5-point scale (1 = strongly disagree, 5 = strongly agree) of the modified RBD scale (α = 0.76, *M* = 3.80, *Mdn* = 3.83, SD = 0.56).

#### Moral Intuition

Moral intuition was measured using the shortened version of the moral foundation questionnaire (MFQ-20). Each of the five moral intuitions was measured using four items. The MFQ-20 consisted of two parts: in part 1, the instruction was included at the top (“when you decide whether something is right or wrong, to what extent are the following considerations relevant to your thinking?), followed by statements for each moral domain (e.g., “…whether or not someone suffered emotionally”). In part 2, the instruction at the top (“please read the following sentences and indicate your agreement or disagreement”) was followed by statements for each moral domain (e.g., “respect for authority is something all children need to learn”). All items were measured based on a 6-point scales (part 1: 0—not at all relevant, 5—extremely relevant; Part 2: 0—strongly disagree, 5—strongly agree) to measure the moral intuition of care (α = 0.71, *M* = 2.98, SD = 0.79), fairness (α = 0.78, *M* = 3.32, SD = 0.81), loyalty (α = 0.66, *M* = 2.61, SD = 0.83), authority (α = 0.76, *M* = 2.49, SD = 0.92), and purity (α = 0.65, *M* = 2.80, SD = 0.77). Subsequently, all MFQ-20 items were averaged into a composite variable to construct the general morality (α = 0.91, *M* = 2.84, SD = 0.69).

#### Fear of COVID

Fear of COVID was measured using the FCV-19S from Ahorsu et al. (24). FCV-19S is a 7-point, 7-item scale that measures the fear of individuals of being infected with COVID-19. Seven items (e.g., “I am most afraid of coronavirus-19”) were translated into Korean and measured on a 7-point scale (1 – strongly disagree, 7 – strongly agree) to measure fear of being infected by COVID-19 in the South Korean context (α = 0.92, *M* = 4.19, SD = 1.26).

#### Health Compliance

A health compliance scale was created to measure COVID-19-related behavioral intentions. This scale comprised eight items on a 7-point scale (1–strongly disagree, 7–strongly agree) to measure four dimensions of COVID-19-related preventive behavioral measures: handwashing, self-isolation, social distancing, and mask wearing. To avoid social desirability bias and ceiling effect (e.g., marking all items to “strongly agree” to questions, such as “do you intend to follow social distancing norms?”), each of the behavioral intentional measures was created for very specific situations. For hand washing, the items were “I will wash my hands more than 30 s every single time I use a restroom no matter how busy I am,” “I will carry hand sanitizers every single time no matter how heavy my pouch is.” For self-isolation, the items were “I will not go to work if I cough or feel the slightest fever,” “I will not meet anybody if I cough or feel the slightest fever.” For social distancing, the items were “I would rather cancel my important schedules if I can't secure a distance of at least 2-meters from other people there,” “I would rather not go to socially important meetings if the meeting place is crowded.” For mask wearing, the items were “I would rather put my mask back on even if I have to drink and eat in a rush,” “I will not take off my mask even for 1 s, no matter how uncomfortable I feel or even when there is nobody around me when I go out.” These eight items were averaged into a mean score to represent a higher score indicating more a preventive behavioral intention against COVID (α = 0.90, *M* = 5.27, SD = 1.03).

### Analysis Plan

#### EPPM Condition Construction

To create EPPM conditions (danger control condition, fear control condition) as independent variables, we adopted the median-split technique, based on the median scores (*Mdn*_*efficacy*_ = 4.50, *Mdn*_*threat*_ = 3.83). Based on the combination of high/low efficacy and threat, contrast coding was used: LE/HT (fear control) was coded as −1 (*n* = 272), either low efficacy/low threat or high efficacy/high threat was coded as 0 (*n* = 895), and HE/LT (danger control) was coded as +1 (*n* = 333).

#### Hypotheses and RQ Testing

To test the hypotheses and RQs, we used Hayes (25). PROCESS Macro 3.5.3 downloaded from processmacro.org website (model = 1, bootstrap = 5,000, moderation conditioning values = −1, 0, +1 SD). For hypotheses testing, each hypothesis (H1–H6) was tested with a combination of RQ1 using PROCESS MACRO v.3.5.3 model 1. The results for all analyses are shown in Table 1.

**Table 1:** Summary of PROCESS v.3.5.3 moderation testing (model = 1, bootstrap = 5,000, conditional effects = −1 SD, *M*, and +1 SD).

	***B* (*S.E*.)**	***p***	**95% *C.I*.**	**Model fit**
**Y: Fear of COVID**				
Self-efficacy (X)	0.32 (0.26)	0.21	−0.18, 0.83	*R^2^* = 0.17,
Morality (w)	1.05 (0.14)	0.00^***^	0.78, 1.33	*p* < 0.001,
Self-efficacy (X) × Morality (w)	−0.20 (0.09)	0.02^*^	−0.38, 0.03	*F* = 99.14
**Y: Health compliance**				
Self-efficacy (X)	0.88 (0.21)	0.00^***^	0.47, 1.30	*R^2^* = 0.16,
Morality (w)	0.66 (0.11)	0.00^***^	0.43, 0.88	*p* < 0.001,
Self-efficacy (X) × Morality (w)	−0.14 (0.07)	0.045^*^	−0.29, −0.003	*F* = 95.77
**Y: Fear of COVID**				
Threat (X)	−0.25 (0.24)	0.30	−0.73, 0.22	*R^2^* = 0.27,
Morality (w)	0.02 (0.13)	0.91	−0.24, 0.27	*p* < 0.001,
Threat (X) × Morality (w)	0.39 (0.08)	0.00^***^	0.22, 0.55	*F* = 184.90
**Y: Health compliance**				
Threat (X)	0.59 (0.21)	0.006^**^	0.17, 1.00	*R^2^* = 0.16,
Morality (w)	0.48 (0.12)	0.00^***^	0.25, 0.71	*p* < 0.001,
Threat (X) × Morality (w)	−0.04 (0.07)	0.60	−0.18, 0.11	*F* = 93.47
**Y: Fear of COVID**				
EPPM (X) (-1: fear control, +1: danger control)	0.23 (0.19)	0.21	−0.13, 0.60	*R^2^* = 0.27,
Morality (w)	0.70 (0.04)	0.00^***^	0.62, 0.78	*p* < 0.001,
EPPM (X) × Morality (w)	−0.31 (0.06)	0.00^***^	−0.43, −0.19	*F* = 186.76
**Y: Health compliance**				
EPPM (X) (-1: fear control, +1: danger control)	0.21 (0.17)	0.21	−0.12, 0.54	*R^2^* = 0.11,
Morality (w)	0.49 (0.04)	0.00^***^	0.42, 0.56	*p* < 0.001,
EPPM (X) × Morality (w)	−0.07 (0.06)	0.21	−0.18, 0.04	*F* = 60.26

* *p < 0.05*,

** *p < 0.01*,

*** *p < 0.001*.

## Results

### Descriptive Statistics

Before testing our hypotheses and RQs, we conducted descriptive statistical analyses for 1,500 samples, with gender (male−763 and female−737) age (below 20–292, 30–272, 40–329, 50–342, and above 60–265), the prevalence of any respiratory symptoms (1,337 had not experienced in the past year), median monthly income (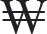
4 million and 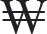
5 million–about $4,000–$5,000 in the U.S.), marital status (married−896), education (high school or below−297, college or below−1,056, above graduate school−147), and political orientation (conservative−270, neutral−869, liberal−361).

#### Main Effects of Self-Efficacy

Hypothesis 1 predicted a negative main effect of self-efficacy on fear of COVID-19. The PROCESS MACRO analysis results did not reveal any main effect of self-efficacy condition on fear (*B* = 0.32, *SE* = 0.26, *p* = 0.21, 95% *CI* = −0.18, 0.83).

Hypothesis 2 predicted a positive main effect of self-efficacy on health compliance. The PROCESS MACRO analysis results revealed that perceived self-efficacy had a positive main effect on health compliance (*B* = 0.88, *SE* = 0.21, *p* < 0.001, 95% *CI* = 0.47, 1.30).

#### The Main Effect of Threat

Hypothesis 3 predicted a positive main effect of threat on fear of COVID-19. The PROCESS MACRO analysis results did not reveal any main effect of threat condition on fear (*B* = −0.25, *SE* = 0.24, *p* = 0.30, 95% *CI* = −0.73, 0.22).

Hypothesis 4 predicted a negative main effect of threat on health compliance. The PROCESS MACRO analysis results revealed a positive main effect of threat condition on health compliance (*B* = 0.59, SE = 0.21, *p* < 0.01, 95% CI = 0.17, 1.00), which was in the opposite direction of H4.

#### EPPM Effect on Fear of COVID

Hypothesis 5 tested the effect of EPPM on fear of COVID-19 so that participants in fear control processing would show greater fear compared to participants in danger control processing. The PROCESS MACRO analysis results did not reveal any interaction between self-efficacy and threat on fear (*B* = 0.23, SE = 0.19, *p* = 0.21, 95% CI = −0.13, 0.60); however, morality moderated the interaction between EPPM conditions and fear (*B* = −0.31, SE = 0.06, *p* < 0.001, 95% CI = −0.43, −0.19). These results suggest that the EPPM effect on fear of COVID was fully moderated by morality (refer to Tables 1, 2 and RQ1 testing below).

**Table 2:** Conditional effects morality as a moderator.

**Moderator levels (w = morality)**	**Effect**	***B* (*S.E*.)**	***p***	**95% *C.I***.
Mean−1 S.D.	Self-efficacy → Fear of Covid	−0.11 (0.09)	0.18	−0.28, 0.05
Mean	Self-efficacy → Fear of Covid	−0.25 (0.06)	0.00^***^	−0.37, −0.14
Mean +1 S.D.	Self-efficacy → Fear of Covid	−0.39 (0.09)	0.00^***^	−0.56, −0.23
Mean−1 S.D.	Self-efficacy → Health compliance	0.57 (0.07)	0.00^***^	0.44, 0.71
Mean	Self-efficacy → Health compliance	0.48 (0.05)	0.00^***^	0.38, 0.57
Mean+1 S.D.	Self-efficacy → Health compliance	0.39 (0.07)	0.00^***^	0.24, 0.51
Mean−1 S.D.	Threat → Fear of COVID	0.58 (0.08)	0.00^***^	0.42, 0.74
Mean	Threat → Fear of COVID	0.84 (0.06)	0.00^***^	0.73, 0.95
Mean +1 S.D.	Threat → Fear of COVID	1.10 (0.08)	0.00^***^	0.95, 1.26
Mean−1 S.D.	EPPM (-1: fear, +1: danger) → Fear of COVID	−0.43 (0.06)	0.00^***^	−0.56, −0.31
Mean	EPPM (-1: fear, +1: danger) → Fear of COVID	−0.64 (0.04)	0.00^***^	−0.73, −0.56
Mean +1 S.D.	EPPM (-1: fear, +1: danger) → Fear of COVID	−0.85 (0.06)	0.00^***^	−0.97, −0.74

*** *p <0.001*.

#### EPPM Effect on Health Compliance

Hypothesis 6 tested the EPPM effect on health compliance so that participants in danger control processing would show higher health compliance than those in fear control processing. The PROCESS MACRO analysis results did not reveal any interaction between perceived self-efficacy and threat on health compliance (*B* = 0.21, SE = 0.17, *p* = 0.21, 95% CI = −0.12, 0.54).

### RQs

#### RQ1: Moderating Effects of Morality

Research question 1 addressed whether morality can moderate the relationship between self-efficacy, threat, fear, and health compliance. The moderating effects of general morality were tested through H1–H6 (refer to Tables 1, 2).

First, morality moderated the relationship between self-efficacy and fear of COVID-19 in medium morality (*M*) and high morality (*M* +1 SD). Specifically, self-efficacy significantly negatively predicted fear of Covid-19 in medium morality (*B* = −0.25, *p* < 0.001, 95% CI = −0.37, −0.14) and high morality (*B* = −0.39, *p* < 0.001, 95% CI = −0.56, −0.23); the effect size of self-efficacy on fear of COVID-19 was stronger in high morality.

Next, morality moderated the relationship between self-efficacy and health compliance in all morality conditions. Specifically, self-efficacy significantly and positively predicted health compliance in low (*B* = 0.57, *p* < 0.001, 95% CI = 0.44, 0.71), medium (*B* = 0.48, *p* < 0.001, 95% CI = 0.38, 0.57), and high (*B* = 0.39, *p* < 0.001, 95% CI = 0.24, 0.51) morality; the effect size of self-efficacy on health compliance decreased as morality increased.

Third, morality moderated the relationship between perceived threat and fear of COVID-19 in all morality conditions. Specifically, perceived threat significantly and positively predicted fear in low (*B* = 0.58, *p* < 0.001, 95% CI = 0.42, 0.74), medium (*B* = 0.84, *p* < 0.001, 95% CI = 0.73, 0.95), and high (*B* = 1.10, *p* < 0.001, 95% CI = 0.95, 1.26) morality; the effect size of perceived threat on fear of COVID increased as morality increased.

Finally, morality moderated the relationship between EPPM interaction and fear of COVID-19 in all morality conditions. Participants in the danger control processing condition were more likely to control fear, compared to participants in the fear control processing condition in low (*B* = −0.43, *p* < 0.001, 95% CI = −0.56, −0.31), medium (*B* = −0.64, *p* < 0.001, 95% CI = −0.73, −0.56), and high morality conditions (*B* = −0.85, *p* < 0.001, 95% CI = −0.97, −0.74). This showed the EPPM effect on fear of COVID-19, which was in line with H5. In addition, the EPPM effect became stronger as morality increased, suggesting that the fear contrast between participants in fear control processing and participants in danger control processing was greater in the higher morality condition.

#### RQ2: Morality, Fear, and Health Compliance

Correlation analyses were conducted to test RQ2, which addressed whether there are relationships between moral intuition, fear of COVID-19, and health compliance behavioral intention. It was found that moral intuition and fear of COVID were significantly related to each other, with *r* (1,498) = 0.39, *p* < 0.001. Also, it was found that moral intuition and health compliance were significantly related to each other, with *r* (1,498) = 0.33, *p* < 0.001.

## Discussion

This study aimed to (a) apply EPPM in the COVID-19 pandemic context, and (b) investigate the role of moral intuition as a moderator to expand EPPM. The current study had several interesting findings.

First, our study showed that the EPPM hypotheses did not show significant results. Specifically, H1, H3, H5, and H6 were found to be non-significant. Although the results of our study showed non-significant results with EPPM hypotheses, this does not mean EPPM is not appropriate to be applied in the Korean COVID-19 pandemic context. This is due to two reasons: first, previous studies still suggested that EPPM can be applied in the East Asian COVID-19 context (3, 4, 9). Second, non-significant results of our study (e.g., H1, H3, H5, and H6) can be still explained in the EPPM context. That is, the current study measured self-efficacy and fear with Witte's (23). RBD scale and adopted median split technique to create EPPM condition variables; notably, the mean values of self-efficacy of 1,500 participants were 4.42 with a median value of 4.50, which was extremely high given that RBD scale is 5-point scale. In other words, this could be the case that both 272 participants in LE/HT conditions and 333 participants in HE/LT conditions were mostly going through danger control processing, instead of fear control processing. This interpretation can make sense with the unexpected finding of H4 that showed a significant positive effect of threat on health compliance, which was in opposite direction. These findings altogether suggest two things: (a) future COVID-19 EPPM studies should consider other ways to prevent the ceiling effect when measuring self-efficacy or fear, and (b) COVID-19 and EPPM can make more sense when other moderating variables, such as morality are adopted.

Moreover, statistical test results with morality as a moderator in EPPM showed that the processing of COVID-19-related messages in the self-efficacy or fear framework can be understood better with the extension of morality. Specifically, our study found that morality moderated the main effects of self-efficacy or perceived threat; morality also moderated EPPM interaction on fear of COVID. Notably, the moderation of morality in the relationship between perceived threat and fear of COVID-19 showed that the effect size of perceived threat increased as morality increased from low morality (*B* = 0.58) to medium (*B* = 0.84) and high morality (*B* = 1.10). Similarly, moderation of morality in the relationship between EPPM interaction and fear of COVID-19 revealed that participants in danger control processing showed less fear than participants in fear control processing did. The contrast of fear between fear control and danger control processing was greater, as the morality increased from low (*B* = −0.43) to medium (*B* = −0.64) and high morality (*B* = −0.85). This suggests that people might think of the threat of being infected by COVID-19 as a moral issue based on the moral intuitions of care, fairness, loyalty, authority, and purity, and such moral thinking moderates the effects on fear.

Finally, the moderation of morality in the relationship between self-efficacy and health compliance behavioral intention showed that health compliance intention decreased as morality increased. Notably, morality decreased COVID-19-related behavioral intentions; this effect could be due to moral licensing arguments [(26), p. 346]. The moral licensing argument suggests that past moral actions of individuals could cause them to take morally dubious actions in the future (e.g., “I have been acting well, so one mistake won't be too bad”). The MFQ-20 measured general moral beliefs, and it is possible that participants were primed to think about past moral deeds. In this sense, participants with higher morality might have felt less inclined to follow COVID-19-related behaviors in the future while thinking about how they had already been acting well in the COVID-19 pandemic situation. In this case, future EPPM studies should consider examining the moral licensing effect in the COVID-19 context.

Our study has several implications. First, we tested the EPPM model in the COVID-19 pandemic context in Korea. We found that EPPM could be useful in understanding how people process COVID-19-related health messages. In addition, the moderation of moral intuition in the EPPM showed that morality could serve as an important variable that can be considered in future studies on COVID-19 or other pandemics. Our findings suggest that people can consider COVID-19 as a social and moral issue that involves protecting others. Therefore, our study implies that researchers, health practitioners, and government officials should also consider social, moral aspects of COVID-19 and individual health.

## Limitations

Although our study had several useful findings for future studies, it has a few limitations as well. First, the overall mean score of participants for perceived self-efficacy was too high (*M* = 4.42); despite our efforts to suppress ceiling effects by adopting the median split technique, it could not have been completely prevented. Future studies should consider other ways to avoid ceiling effects. Second, our study included only participants from South Korea. Future studies can include other East Asian countries, such as Japan and China, to examine whether East Asian countries, in general, can perceive pandemic disease as a moral issue. Third, there was a limitation in the health compliance measures we used. Although eight items we created show high reliability of α = 0.90, our study did not thoroughly validate these measures with other means (e.g., confirmatory factor analysis). Also, although these items were created to avoid the ceiling effect, our data showed that the mean value was still high with *M* = 5.27. Still, the results of our hypotheses testing (H2 and H4) and moderation testing show that our measure can be still useful. Future studies can include an improved measure of health compliance in the COVID-19 context.

## Conclusion

In conclusion, we tested the EPPM model in the COVID-19 context in South Korea and introduced moral intuition as a moderator. Our study showed that morality could serve as a new important variable in the processing of pandemic-disease messages, which can expand EPPM theory.

## Data Availability Statement

The raw data supporting the conclusions of this article will be made available by the authors, without undue reservation.

## Ethics Statement

The studies involving human participants were reviewed and approved by the Hallym University Institutional Review Board. The patients/participants provided their written informed consent to participate in this study.

## Author Contributions

All authors listed have made a substantial, direct and intellectual contribution to the work, and approved it for publication.

## Funding

This work was supported by the Ministry of Education of the Republic of Korea and the National Research Foundation of Korea (NRF-2018S1A3A2074932).

## Conflict of Interest

The authors declare that the research was conducted in the absence of any commercial or financial relationships that could be construed as a potential conflict of interest.

## Publisher's Note

All claims expressed in this article are solely those of the authors and do not necessarily represent those of their affiliated organizations, or those of the publisher, the editors and the reviewers. Any product that may be evaluated in this article, or claim that may be made by its manufacturer, is not guaranteed or endorsed by the publisher.

## References

[1] Witte K. Fear control and danger control: A test of the extended parallel process model (EPPM). Commun Monographs. (1994) 61:113–34. 10.1080/03637759409376328

[2] Jahangiry L Bakhtari F Sohrabi Z Reihani P Samei S Ponnet K. Risk perception related to COVID-19 among the Iranian general population: an application of the extended parallel process model. BMC Public Health. (2020) 20:1–8. 10.1186/s12889-020-09681-7 33076875 PMC7570396

[3] Lin HC Chen CC. Disease prevention behavior during the COVID-19 pandemic and the role of self-esteem: an extended parallel process model. Psychol Res Behav Manag. (2021) 14:123–35. 10.2147/PRBM.S291300 33603513 PMC7882452

[4] Zhao S Wu X. From information exposure to protective behaviors: investigating the underlying mechanism in COVID-19 outbreak using social amplification theory and extended parallel process model. Front Psychol. (2021) 12:e631116. 10.3389/fpsyg.2021.631116 34113280 PMC8185043

[5] Kim ES Chung JB. Korean mothers' morality in the wake of COVID-19 contact-tracing surveillance. Soc Sci Med. (2021) 270:1–8. 10.1016/j.socscimed.2021.113673 33453628 PMC7833862

[6] Graham J Haidt J Koleva S Motyl M Iyer R Wojcik SP. Chapter Two – Moral Foundations Theory: The Pragmatic Validity of Moral Pluralism. In: Devine P, Plant A, editors. Advances in Experimental Social Psychology. Vol. 47. Academic Press (2013). p. 55–130. 10.1016/B978-0-12-407236-7.00002-4

[7] Graham J Nosek BA Haidt J Iyer R Koleva S Ditto PH. Mapping the moral domain. J Pers Soc Psychol. (2011) 101:366–85. 10.1037/a0021847 21244182 PMC3116962

[8] Leventhal H. Findings and theory in the study of fear communications. In: Berkowitz L, editor. Advances in Experimental Social Psychology. New York, NY: Academic (1970).

[9] Yang J Wu X Sasaki K Yamada Y. No significant association of repeated messages with changes in health compliance in the COVID-19 pandemic: a registered report on the extended parallel process model. PeerJ. (2021) 9:e11559. 10.7717/peerj.11559 34141490 PMC8180189

[10] Barnett DJ Balicer RD Thompson CB Storey JD Omer SB Semon NL. Assessment of local public health workers' willingness to respond to pandemic influenza through application of the extended parallel process model. PLoS ONE. (2009) 4:6365. 10.1371/journal.pone.0006365 19629188 PMC2711331

[11] Lithopoulos A Liu S Zhang CQ Rhodes RE. Predicting physical distancing in the context of COVID-19: A test of the extended parallel process model among Canadian adults. Canad Psychol. (2021) 62:56–64. 10.1037/cap0000270

[12] Meadows CZ Meadows CW Tang L. The CDC and state health department facebook messages: an examination of frames and the extended parallel processing model. Commun Stud. (2020) 71:740–52. 10.1080/10510974.2020.1819839

[13] Shirahmadi S Seyedzadeh-Sabounchi S Khazaei S Bashirian S Miresmæili AF Bayat Z. Fear control and danger control amid COVID-19 dental crisis: application of the extended parallel process model. PLoS ONE. (2020) 15:e237490. 10.1371/journal.pone.0237490 32790730 PMC7425864

[14] Woyessa AH Oluma A Palanichamy T Kebede B Abdissa E Labata BG. Predictors of health-care workers' unwillingness to continue working during the peak of COVID-19 in Western Ethiopia: an extended parallel-process model study. Risk Manag Healthc Policy. (2021) 14:1165–73. 10.2147/RMHP.S288003 33762859 PMC7982702

[15] Chan EY. Moral foundations underlying behavioral compliance during the COVID-19 pandemic. Pers Individ Dif. (2021) 171:1–10. 10.1016/j.paid.2020.110463 33106715 PMC7577686

[16] Rozin P Lowery L Imada S Haidt J. The CAD triad hypothesis: A mapping between three moral emotions (contempt, anger, disgust) and three moral codes (community, autonomy, divinity). J Pers Soc Psychol. (1999) 76:574–86. 10.1037/0022-3514.76.4.574 10234846

[17] Triandis HC. Individualism-collectivism and personality. J Pers. (2001) 69:907–24. 10.1111/1467-6494.696169 11767823

[18] Grizzard M Ahn C. Morality personality: perfect deviant selves. In Banks J, editor. Avatar, Assembled: The Social and Technical Anatomy of Digital Bodies. New York, NY: Peter Lang (2017).

[19] Haidt J. The moral emotions. In: Davidson RJ, Scherer KR, Goldsmith HH, editors. Handbook of Affective Sciences. Oxford: Oxford University Press (2003).

[20] Haidt J Joseph C. The moral mind: How five sets of innate intuitions guide the development of many culture-specific virtues, and perhaps even modules. In: Laurence CP, Stich SS, editors. Evolution and Cognition. The Innate Mind Vol. 3. Foundations and the Future. Oxford: Oxford University Press (2008).

[21] Harper CA Satchell LP Fido D Latzman RD. Functional fear predicts public health compliance in the COVID-19 pandemic. Int J Mental Health Addict. (2020) 27:1–14. 10.1007/s11469-020-00281-5 32346359 PMC7185265

[22] Qian K Yahara T. Mentality and behavior in COVID-19 emergency status in Japan: Influence of personality, morality and ideology. PLoS ONE. (2020) 15:e235883. 10.1371/journal.pone.0235883 32649687 PMC7351180

[23] Witte K. Predicting risk behaviors: Development and validation of a diagnostic scale. J Health Commun. (1996) 1:317–42. 10.1080/108107396127988 10947367

[24] Ahorsu DK Lin CY Imani V Saffari M Griffiths MD Pakpour AH. The fear of COVID-19 scale: development and initial validation. Int J Ment Health Addict. (2020) 27:1–9. 10.1007/s11469-020-00270-8 32226353 PMC7100496

[25] Hayes AF. Introduction to Mediation, Moderation, and Conditional Process Analysis: A Regression-Based Approach (2nd Edition). New York, NY: Guilford publications (2017).

[26] Merritt AC Effron DA Monin B. Moral self-licensing: When being good frees us to be bad. Soc Personal Psychol Compass. (2010) 4:344–57. 10.1111/j.1751-9004.2010.00263.x

